# Decoding Emotion in Drug Abusers: Evidence for Face and Body Emotion Recognition and for Disgust Emotion

**DOI:** 10.3390/ejihpe12090099

**Published:** 2022-09-17

**Authors:** Natale Salvatore Bonfiglio, Roberta Renati, Gabriella Bottini

**Affiliations:** 1 Department of Pedagogy, Psychology, Philosophy, University of Cagliari, 09123 Cagliari, Italy; 2 Noah srl, 27100 Pavia, Italy; 3 Department of Brain and Behavioral Science, University of Pavia, 27100 Pavia, Italy; 4 Cognitive Neuropsychology Center, ASST Grande Ospedale Metropolitano Niguarda of Milan, 20162 Milano, Italy; 5 NeuroMi, Milan Center for Neuroscience, 20126 Milano, Italy

**Keywords:** emotional processing, disgust, cocaine dependence, alcohol dependence, face, body

## Abstract

*Background:* Different drugs damage the frontal cortices, particularly the prefrontal areas involved in both emotional and cognitive functions, with a consequence of decoding emotion deficits for people with substance abuse. The present study aimed to explore the cognitive impairments in drug abusers through facial, body and disgust emotion recognition, expanding the investigation of emotions processing, measuring accuracy and response velocity. *Methods:* We enrolled 13 addicted to cocaine and 12 alcohol patients attending treatment services in Italy, comparing them with 33 matched controls. Facial emotion and body posture recognition tasks, a disgust rating task and the Barrat Impulsivity Scale were included in the experimental assessment. *Results:* We found that emotional processes are differently influenced by cocaine and alcohol, suggesting that these substances impact diverse cerebral systems. *Conclusions:* Drug abusers seem to be less accurate on elaboration of facial, body and disgust emotions. Considering that the participants were not cognitively impaired, our data support the hypothesis that emotional impairments emerge independently from the damage of cognitive functions.

## 1. Introduction

Drug users affect several neural systems at the subcortical and cortical levels, causing cognitive and emotional impairments [1,2,3,4]. Drugs use and dependence, comprising recreational use only, impair different regions of the prefrontal cortex [5].

However, there are differences in the induced impairments depending on the diverse drugs used alone or at the same time [6], and there are also relevant commonalities. Excitant drugs, such as amphetamine and cocaine, as well as in alcohol, for example, show altered functioning of the prefrontal cortex (PFC) of users [7,8], particularly relating to the medial prefrontal cortex [9]. Recreational use of cannabis and psychostimulants have also been associated with mild executive deficits [10,11,12,13,14]. Therefore, the PFC cerebral area is crucial in self-monitoring and self-control: the more the exposure, the greater the level of craving [15,16,17,18,19] and dependence [20]. Furthermore, prefrontal cortex dysfunction, known to be primarily involved in motivation and decision-making processes, prevents good compliance with any treatment [21,22,23].

There are several studies on the cognitive disorders caused by drugs abuse and the emotional decoding of drugs abusers’ competence, and emotion recognition from faces is one of the most extensively investigated areas in individuals with alcohol use disorder. Most studies identified impairments in the decoding of emotions from faces [21,22,23,24,25,26,27,28,29,30,31,32] and in facial emotion recognition in alcohol-user patients [33,34,35,36,37]. Many other studies with cocaine users have also revealed impairment in their ability to identify basic facial affect expressions [38,39,40,41,42]. However, a few studies have found specific alterations in fear and anger decoding processing from faces in cocaine users [43,44,45]. Moreover, in polysubstance users, recognition of these emotions was negatively correlated with cocaine use intensity [46].

Variability in the accuracy of recognition of emotions such as fear has often been correlated with indices of intelligence and the tendency to compensate for dysfunctional brain networks or damaged brain tissue following a pre-morbid tendency, revealing a potential relationship between IQ and fear recognition in cocaine users [43]. However, impairment in fear recognition has been observed in the literature in habitual cocaine users and is mainly related to the frequency of substance use [44]. In addition, lower gray matter volumes in specific cortical and subcortical regions support the idea of a neural deficit present in cocaine addicts [44], as well as serotonin dysregulation and amygdala dysfunction, which could explain the fear recognition deficits in frequent cocaine users [45].

Many aspects of cognitive dysfunction have also been described in alcoholics, some of which may be a consequence of deficits in cognitive function of the prefrontal areas of the cortex [25] and a cause of reduced neuroplasticity, which may lead to errors in emotion recognition [23]. Such deficits do not seem to improve after a long period of abstention from substance use [34].

Therefore, it is important to focus on the relationship between emotion recognition, with a focus on fear and anger, and cognitive deficiencies resulting from alcohol and cocaine use in individuals with addiction to these substances.

Is also important to consider that, in real life, people detect relevant social signs by decoding not only facial expressions but also emotional body postures. The emotional recognition problem is a crucial issue, as abusers frequently manifest problems in social interaction, due to, for example, confounding or misinterpreting emotions such as fear and anger [47,48,49,50,51,52]. Furthermore, the success of psychosocial or behavioral treatments, such as residential treatments or training based on cue exposure or attentional bias, to induce the maintenance of drug withdrawal mainly relies on the neural regulations of areas such as the PFC, which has a key role in emotional processing and craving [14,16,21,50,53]. Escalation and maintenance of drug dependence are associated with dysregulation of the anatomical structures that are also involved in the emotional circuit [39] and in the anatomical areas associated with awareness of body emotional language, such as the ventromedial prefrontal cortex, insula and anterior cingulate cortex [52].

There is evidence that dependence on alcohol causes not only an overestimation of the intensity of the emotional facial expressions of happiness, anger and disgust [29,31] associated with a poorer recognition of sadness [54], but also difficulties in discriminating anger and disgust [31,55,56]. Indeed, selective alterations in fear recognition have been shown in cocaine and polysubstance psychostimulant abusers [45,46].

At the ontogenetic level, disgust is considered the refusal of, or defense from, potentially harmful food products or contaminants for the individual (core disgust) [57]. However, with the evolution of society, four additional domains have been identified: poor hygiene, inappropriate sexual acts, death and violation of the ideal body or external form, which in turn are linked to the broader domain of moral disgust [57]. Contamination and moral disgust are both relevant elements in substance dependence, considering the modalities to assume different drugs and the recurrent violation of the body, such as drug injection. Identifying selective impairments of emotional decoding may contribute to better focus on different forms of treatment.

Understanding the processes of emotion coding, especially relative to negative emotions, could offer a significant contribution in creating more effective treatments for dependent people. On the psychosocial level, for example, this involves creating more focused psychoeducational activities or individual and/or group activities that focus on roleplay and simulations of relational situations. On a neurocognitive level, the understanding of emotion encoding processes could help to create interventions based on the relationship between emotions and specific cognitive processes. Several interventions are, in fact, based on both assessment and structuring training related to cognitive biases associated with prefrontal areas, such as the *attentional probe*.

In this study, we aimed to investigate emotional decoding in subjects with alcohol and cocaine dependence, including body posture recognition and the processing of disgust, and to explore the ability to express these emotions [58] by measuring both accuracy and velocity in terms of reaction times, as these cognitive competencies are typically associated with the PFC. We believe that our study will provide data concerning (i) the impact of cocaine and alcohol on emotional processing, (ii) the influence of the duration of consumption of both substances on emotional processing and, finally, (iii) the putative influence of cognitive dysfunction related to the prefrontal cortex on emotional competencies.

## 2. Methods

### 2.1. Participants

We enrolled 25 individuals diagnosed with substance dependence (see Table 1 for demographic and clinical features) attending Italy’s government specialist addiction treatment service. Thirteen participants were primarily addicted to cocaine and 12 to alcohol. All were right-handed, and none showed pathological gambling. Eight patients did not have previous treatments, 14 had one treatment, 2 had two treatments, and one had four treatments. One had a mood stabilizer and one anxiolytic pharmacological treatment. Only four individuals were under methadone treatment at the time of the experiment. Inclusion criteria to participate in the study were (i) diagnosis of substance dependence according to the DMS-IV TR criteria, (ii) absence of brain damage due to other diseases (i.e., traumatic brain injury), (iii) absence of sensory or neurological disorders (i.e., blindness, dementia) and (iv) abstinence from the substance in taking at least 55 days before the experiment.

Thirty-three participants were recruited as a control group at the University of Pavia (see Table 1 for demographic features). Inclusion criteria for these subjects were (i) absence of substance abuse/dependence (with the exclusion of drinks; if moderate drinking, on average, less than 10 drinks per week), (ii) absence of brain damage due to other diseases (i.e., traumatic brain injury), (iii) absence of sensory or neurological disorders (i.e., blindness, dementia), (iv) absence of psychiatric or affective disturbances diagnosed by a specialist and (v) absence of drug treatment.

### 2.2. Materials

#### 2.2.1. Facial Emotion Recognition (FER)

The FER is composed of male and female faces taken from the Ekman and Friesen series [59], expressing one of the following emotions at full intensity: fear, sadness, happiness and anger. Surprise was not included in the set, as previous studies reported that even healthy individuals frequently mistake this emotion for fear [60]. Furthermore, disgust has been excluded from obtaining a completely comparable set to the one adopted for postures (see BEAST description below). The task includes 12 displays for each emotion, portrayed by four individuals, leading to 48 trials. Stimuli were presented centrally, interspersed with a fixation cross between trials (1000 ms), and they remained on the screen until the participant’s response. Five labels (corresponding to the four emotions presented on the test and to an “I do not know” option) were shown below the screen and attached to the computer keyboard. Participants had to press the label corresponding to the emotion of the target image with their dominant hand. They had to answer as quickly and accurately as possible, but there was no time limit to complete a trial. The order of images was randomized across participants and within the same subject. We did not provide any feedback on performance accuracy during the experiment. Subjects were instructed to use the “I do not know answer” as few times as possible. The score was calculated as the percentage of correct answers to the total number of stimuli presented.

#### 2.2.2. Body Emotion Recognition (BEAST)

BEAST consisted of male and female whole bodies images expressing one of the following emotions: fear, sadness, happiness and anger. Images were taken from the Bodily Expressive Action Stimulus Test, BEAST [61]. This task includes four displays for each emotion, displayed by four individuals, leading to 48 trials. Stimuli were presented, and responses were recorded with the same procedure as for the FER task. The score was calculated as the percentage of correct answers to the total number of stimuli presented.

#### 2.2.3. Disgust Rating Task (DRT)

This task is composed of pictures depicting fear, sadness, happiness and anger as controls and images displaying disgusting scenes or items [58,62]. Pictures are taken from the International Affective Pictures System [63] and the internet. For disgust, we presented 24 images: 4 images related to food, 4 related to body products, 2 related to animals, 2 related to contamination, 2 related to death, 2 pictures related to envelope violations, and 4 images related to hygiene. Participants were presented with 36 real-life pictures and asked to rate the intensity of the picture. Ratings could range from 1 (not at all disgusting) to 7 (completely disgusting). Participants were asked to press one of the seven possible buttons on the keyboard using the dominant hand as rapidly as possible for each image. Stimuli were presented centrally until the subject’s response was recorded, interspersed with a fixation cross (1000 ms). The score was calculated as the average of correct answers for each category.

#### 2.2.4. Barratt Impulsiveness Scale (BIS)

This self-reported scale [64] is used to measure impulsiveness. It comprises 30 items that yield six first-order factors related to the construct measured (attention, motor impulsiveness, self-control, cognitive complexity, perseverance and cognitive instability) and three second-order factors (attentional, motor and non-planning impulsiveness). The answers are given on a four-point Likert scale (1: Never/Rarely; 2: Sometimes; 3: Often and 4: Almost always/Always). The total score is calculated by summing the scores obtained at the individual first-order subscales. Authors report a mean of 64.11 (SD = 10.07) for the Italian population. An Alpha value of 0.79 has been reported.

#### 2.2.5. Brief Intelligence Test (TIB)

The TIB [65] is the Italian equivalent of the “National Adult Reading Test” [66] and assesses the premorbid intelligent quotient (premorbid IQ). It consists of 54 words, 34 of which have an irregular pronunciation, while the others are control words with a high frequency of use. This instrument has also been adapted for a computer presentation. The participant’s task is to read the word while the experimenter records the answer, according to whether the participant committed an error. Errors of pronunciation or accent are coded with 1, errors of both pronunciation and accent are coded with 2 and no errors are coded as 3. The total number of errors for the irregular words gives the final score. An Alpha value of 0.94 has been reported.

#### 2.2.6. Raven Advanced Progressive Matrices (RAPM)

The RAPM [67] is used to measure nonverbal intelligence and reasoning abilities. It consists of 48 stimuli structured as multiple-choice questions. Items are presented in black ink on a white background and become increasingly difficult as progress is made through each set. For each test item, the subject is asked to identify the missing element that completes a pattern. Twelve items compose the training set, while 36 effective test items are used. Being adapted for a computer presentation, this task requires participants to choose among eight alternative answers. The score was calculated as the number of correct items.

### 2.3. Procedure

Each group was administered the whole battery. The experiment was conducted in a quiet room, with the computer (screen size 16″) positioned on a desk approximately 50 cm from the participant’s eyes. Tasks were administered through dedicated software [Opensesame©, [68], which allowed the collection of reaction time (RT) and accuracy. Participants were required to use their dominant hand to answer by pressing the appropriate key on the computer keyboard. The six tasks were presented in random order. In all tasks, participants were instructed to respond as quickly and as accurately as possible. Only accuracy was recorded in the TIB and in the RAPM, while RT was also collected for the other tests.

### 2.4. Data Analyses

Data were analyzed through Statistical Package for Social Sciences (SPSS) version 19 for WINDOWS.

The Shapiro–Wilks test for normality and visual inspection of histograms and boxplots were used to detect departures from normality on all computed measures. The Leven’s test was used to test equity of variance.

Because the assumptions of homoscedasticity and normality were violated, Bias-corrected bootstrapping (n = 2000) was used to deal with violated assumptions [69].

Data from the DRT, BIS, TIB, RAPM, FER and the BEAST tasks were analyzed through a one-way ANOVA. This procedure was applied to accuracy (percentage of correct answers), RT (average reaction time for correct responses only) and IES (inverse efficiency score).

Demographic variables were compared using a Student *t* Test. Correlation between DRT, BIS, TIB, RAPM, FER and the BEAST tasks and clinical variables were also analyzed.

The gender differences between male/female patients and male/female controls, interaction of both genders in the two groups, onset of drug dependence, the days of abstinence and the type of secondary substance were analyzed through a one-way ANOVA.

The alpha level was set at 0.05. Effect sizes of significant effects are presented as partial eta squared *(η*^2^*_p_*) values.

#### Demographic Variables

This preliminary analysis confirmed no significant differences, neither between controls and dependent individuals with respect to age, gender and education (Table 1) nor for each DRT, FER and RAPM subscale (Table 2).

## 3. Results

No significant differences were found between demographic variables (*p* > 0.05). The one-way ANOVA showed a significant difference for cognitive level between groups considering the RAPM scores, with lower scores reported from cocaine subjects compared with the control group. A significant trend was also found for the BIS score, in which cocaine subjects reported higher impulsivity scores (Table 2).

For accuracy, no differences were found for FER and BEAST (Table 3).

With regards to reaction times, significant differences were found for FER and BEAST (Table 4). A significant trend was found for sadness facial expressions and both for anger and sadness body expression. The post-hoc analysis revealed that cocaine-dependent subjects were slower than controls in the recognition of sadness facial expression, and alcohol-dependent subjects were slower in recognizing both anger and sadness body expression.

With regards to accuracy of DRT, no differences were found (Table 5).

With regards to the reaction times of the DRT, a significant difference was found for food images (Table 6). The post-hoc analysis revealed that alcohol-dependent subjects were slower than controls in responding to disgusting food images.

As regards IES, a significant difference was found using one-way ANOVA for both sadness and happiness of the BEAST task. The post-hoc analysis revealed that alcoholic subjects were impaired in sadness and happiness emotion recognition compared to controls (Table 7).

With regards to substances and gender interactions, a significant difference was found on RTs for both the fear emotion of FER (F_(5,57)_ = 4.12; *p* = 0.022; *η*^2^*_p_* = 0.13) and amputation images of the DRT (F_(5,55)_ = 3.25; *p* = 0.047; *η*^2^*_p_* = 0.11), with higher reaction times reported by females with cocaine dependence. Moreover, a significant trend was also found on RTs regarding the sadness emotion of the FER (F_(5,57)_ = 3.1; *p* = 0.058; *η*^2^*_p_* = 0.11) and both body product images (F_(5,57)_ = 2.9; *p* = 0.062; *η*^2^*_p_* = 0.10) and death images (F_(5,56)_ = 3; *p* = 0.057; *η*^2^*_p_* = 0.11) of the DRT, with higher reaction times reported for females with cocaine dependence.

Considering the onset of drug dependence for the primary substance of dependence, the days of abstinence and the type of secondary substance, we did not find any significant correlation with any of the measures we adopted to explore emotional processing (*p* > 0.05).

## 4. Discussion

This study aimed to detail the emotional profile of individuals with dependence on psychostimulants or depressor drugs by expanding on the recognition of body postures and disgust processing. We found that individuals with dependence on alcohol and cocaine could accurately recognize emotions from the face and the body, regardless of the emotional categories measured. However, weighing accuracy and velocity rules out a trade-off that suggests that these individuals need significantly more time to reach the same accuracy as individuals without dependence. Similarly, individuals with alcohol dependence are generally slower than controls. There are already studies reporting that alcohol abusers need significantly more time than controls “*to answer accurately to questions about the emotional decoding of the emotional facial expression task, regardless of the exposure time of the stimulu, and the type of answer expected*” [29] (p. 39). Based on this observation, using time-limited tasks might also help appreciate significant differences in terms of accuracy. The results for alcohol abusers suggest that the entire emotional system has “slowed down” despite withdrawal from the abuse.

We did not find the same effect in cocaine abusers. Similarly, Woicik and colleagues [42] found that cocaine abusers are not different from controls in facial emotion recognition tasks. Previous studies, testing accuracy in polyabusers, showed that the quantity of drugs used could explain the impairment of emotions in recognizing facial expressions in the use of substances throughout the entire lifespan [46].

However, our data show that dependent subjects were slower in recognizing disgusting stimuli if they belonged to the “food” category and showed higher BIS scores than controls. What we have found agrees with the conclusions of recent studies [70]. However, our results outline a more accurate profile for the different forms of disgust. These findings also agree with previous studies showing a significant impact of cocaine in emotional and inhibition processing [16,46].

One could speculate that prolonged cocaine use affects the striatum, connected through serial and parallel pathways to the basal ganglia and the prefrontal cortex [71,72]; indeed, both structures are involved in food processing. Interestingly, studies have shown that individuals with dependence, regardless of the substance abused, show impairments of the prefrontal cortex [5,73] and that the anatomical structures of the reward system appear to be dysfunctional in both subjects with food intake dysregulation and cocaine abuse [74]. This cerebral structure is crucial in monitoring executive functions and emotional processes [51,74,75]. Both components are relevant in complex behaviors such as decision making, which is central for compliance with treatments and the maintenance of drug withdrawal [3,15,16,18]. We speculate that emotional processing impairments that reduce the ability to decode social signs (relevant for interaction and communication) also negatively affect drug abuse treatment [2,76]. Very little is known about emotional processing and dependence; in particular, the results on facial emotion recognition are mixed [55].

Disgust has evolved as a “behavioral immune system” to help people defend against specific environmental threats, classified as pathogenic (e.g., spoiled food, feces, disease), sexual (sexually transmitted diseases) and moral (moral transgressions) [77]. Disgust is associated with a growing concern about health/cleanliness concepts and health prevention strategies [78]. It has been argued that alterations in drive states may reduce sensitivity to disgust and could potentially lead to increased risk-taking (having unprotected sex, consuming spoiled food) [79]. It is conceivable that altered drive states in cocaine-dependent individuals could lead to reduced disgust sensitivity. Similar effects have been observed, for example, when individuals in a high hunger state show less disgust activity when they have been exposed to images of unpleasant food [79]. It is possible that the high levels of impulsivity and the long period of abstinence reported in cocaine-addicted individuals of our group have increased craving and the need to use substance, that could explain the reduced disgust sensitivity. The triggering of disgust activates parts of the brain (particularly the anterior insula) that are also activated by central disgust, but the anterior insula is not uniquely associated with disgust (and vice versa) [57]. The insula has been shown to also be a critical neural substrate for craving in addiction [80]. Future research could better investigate the relationship between disgust and craving in cocaine-addicted subjects.

We found a significant difference for the groups concerning RTs for anger and sadness face expression and sadness body expression, suggesting that individuals with a dependence on alcohol and cocaine are slower in recognition of these emotions expressed by the face and the body than controls. However, as no main effect emerged for accuracy, these findings could only represent a trade-off between speed and accuracy. This effect could be plausible considering that no time limit has been assigned for these tasks. The inverse efficiency score (IES) [81] is a combined index accounting for speed and error rates. The IES is calculated by dividing the RT value by the accuracy score. Thus, in the current study, such a conversion allows controlling of the impact of accuracy on the speed of responses. This measure provides a more reliable and stable measure of emotional impairment when recognition is intact. Considering IES, we can imagine that impairment for sadness and happiness emotion recognition are to be attributed to prolonged alcohol use.

We also found that cocaine-addicted females showed slower response times compared to males for the facial expression of and for body product and amputation images. This may simply reflect the smaller sample of females in our study [44].

## 5. Conclusions

The aim of this study was to investigate emotional decoding, as a means of face and body expression, including the processing of disgust, in patients using cocaine and alcohol. Both accuracy and velocity in terms of reaction times were measured, as these cognitive competencies are typically associated with the prefrontal cortex. We found that alcohol users are generally slower in recognizing emotions, and that dependent subjects are slower in recognizing disgusting stimuli if they belong to the “food” category.

Although preliminary, we believe that this study provides useful information for more focused clinical settings. However, these results should be interpreted with caution before being applied. The results offer confirmation of previous data showing an emotion recognition decoding deficit in cocaine addicts and alcoholics, strongly suggesting a specificity of this deficit for emotions. This may have implications for clinical and treatment situations. Indeed, deficits in the assessment of emotional facial expressions have been shown to be related to interpersonal problems and may be linked to relapse risk [24]. The results that emerged in our study could help in implementing new features for a treatment program focusing on motivation, communication, interaction and ability to cope with dependence problems [82,83,84].

### Limitations and Future Directions

The present findings provide the basis for several directions for future research. More in-depth studies using a resonance imaging technique could better analyze which areas and networks are involved in response to emotions like disgust. It is also possible that the differences in the accuracy of disgust images can also be attributed to moral differences, conveyed by social and cultural context and individual upbringing.

Despite the important strengths of the present study, future research should consider addressing some important limitations, such as the low number of subjects involved in the study and the non-balanced frequency distribution of the participants in relation to gender [85] and substance used. Moreover, the absence of any personal features (such as self-determination) that may be lacking in other subjects with the same substance addiction may be considered. In addition, more specific objective tests and test batteries should be used to evaluate neuropsychological deficits. These limits are, however, linked to the characteristics of the subjects who access addiction care services. Moreover, we distinguished and compared participants based on their primary dependence, but future works could deeply assess the influence of the secondary abused substance on emotional decoding processing. Finally, the lack of information about psychiatric condition (like anxiety or depression) could be a possible confounding factor for the results interpretation [86].

## Figures and Tables

**Table 1:** Age, education, frequency of use, age at first use for the primary substance and days of abstinence and frequency of use depicted as a group average and standard deviation. All the other variables show the number of subjects for each category.

	Patients	Controls	Test	Statistics	*p* Value
	n = 25	n = 33			
Age	41.3	39.8	*t* Test	−0.5	0.62
	±11.3	±11.4			
Education	10.5	11.7	*t* Test	1.16	0.25
	±3.4	±4.5			
Gender	19 M	25 M	*X* ^2^	0.0004565	0.98
	6 F	8 F			
Poly-subtsance abusers	13				
	First Cocaine	13	binomial	0.52 *	0.98
	First Alchool	12		0.48 *	0.99
	Secondary Heroin	4	binomial	0.31 *	0.27
	Secondary Cocaine	3		0.23 *	0.092
	Secondary Alcohol	4		0.31 *	0.27
	Secondary THC	2		0.15 *	0.022
Age first use primary substance		21.8			
		±8.7			
Days of abstinence		55			
Frequency of use before treatment (days per week)		5.2			

* = portion tested against value 0.05, Legend: M = males, F = females.

**Table 2:** Statistics, mean and standard deviation for cognitive and impulsivity variables.

		*Mean*	*SD*	*Bootstrapped SE*	*Bootstrapped CI*		*F*	*Sig.*	*η^2^_p_*
*RAPM*	control	22.94	10.07	1.71	19.5	26.3	5.35	0.008	0.163
	alcohol	19.92	11.54	3.20	13.8	26.8			
	cocaine	12.42	3.85	1.10	10.4	14.6			
*TIB*	control	97.49	15.83	2.73	91.6	102.6	1.56	0.218	0.054
	alcohol	12.42	3.85	5.41	80.4	102.1			
	cocaine	19.92	11.54	4.78	78.6	97.5			
*BIS*	control	62.55	8.478	1.42	59.75	65.42	3.15	0.051	0.103
	alcohol	66.00	4.452	1.27	63.45	68.50			
	cocaine	69.38	11.110	3.10	63.50	75.23			

*Note*: bootstrap estimation was used to derive 95% confidence intervals. Raven Advanced Progressive Matrices (RAPM); Brief Intelligence Test (TIB); Barratt Impulsiveness Scale (BIS); Standard Deviation (SD); Standard Error (SE); Confidence Interval (CI).

**Table 3:** Statistics, mean and standard deviation of accuracy for face and body emotional tasks.

			*Mean*	*SD*	*Bootstrapped SE*	*Bootstrapped CI*		*F*	*SIG.*	*η* ^2^ *_P_*
***FER***	anger	control	72.73	20.86	3.61	65.63	80.00	1.469	0.239	0.051
		cocaine	82.69	11.52	3.28	76.52	89.10			
		alcohol	77.08	14.27	4.11	68.75	85.26			
	fear	control	69.70	13.31	2.23	65.23	74.11	1.971	0.149	0.067
		cocaine	72.44	7.89	2.21	68.33	77.08			
		alcohol	63.19	12.03	3.45	56.25	69.64			
	sadness	control	75.50	24.38	4.13	67.29	83.59	1.583	0.215	0.054
		cocaine	83.33	20.13	5.45	71.80	92.86			
		alcohol	87.50	13.06	3.85	79.55	94.44			
	happiness	control	93.94	15.35	2.62	88.02	98.33	0.566	0.571	0.020
		cocaine	96.15	7.31	2.05	91.67	100.00			
		alcohol	88.89	28.50	7.93	71.11	99.24			
***BEAST***	anger	control	88.80	15.93	2.83	83.01	93.94	0.052	0.950	0.002
		cocaine	87.18	17.22	4.82	77.27	96.07			
		alcohol	87.88	14.12	4.31	79.17	95.83			
	fear	control	89.25	13.64	2.41	84.29	93.49	0.638	0.532	0.024
		cocaine	89.74	9.71	2.78	84.17	95.14			
		alcohol	84.72	13.22	3.65	77.08	91.67			
	sadness	control	89.06	10.68	1.79	85.48	92.50	1.020	0.367	0.036
		cocaine	83.33	15.21	4.18	74.57	91.67			
		alcohol	88.89	14.36	4.12	80.21	96.43			
	happiness	control	84.70	14.98	2.59	79.19	89.54	0.989	0.378	0.035
		cocaine	76.92	17.40	4.71	67.86	86.11			
		alcohol	83.33	21.02	5.94	70.83	94.27			

*Note*: bootstrap estimation was used to derive 95% confidence intervals. Facial Emotion Recognition (FER); Body Emotion Recognition (BEAST); Standard Deviation (SD); Standard Error (SE); Confidence Interval (CI).

**Table 4:** Statistics, mean and standard deviation of reaction times for face and body emotional tasks.

			*Mean*	*SD*	*Bootstrapped SE*	*Bootstrapped CI*		*F.*	*SIG.*	*η* ^2^ *_P_*
***FER***	anger	control	2236.98	1260.45	222.76	1837.54	2712.04	2.032	0.141	0.069
		cocaine	2981.11	1595.84	445.81	2172.96	3919.00			
		alcohol	2838.40	863.11	251.54	2353.88	3359.33			
	fear	control	2307.41	879.84	155.75	2009.34	2636.43	2.850	0.066	0.094
		cocaine	2994.22	1545.16	428.77	2189.67	3865.87			
		alcohol	3052.02	1237.99	351.52	2389.76	3751.83			
	sadness	control	2074.67	886.82	151.33	1807.35	2405.56	5.672	0.006	0.171
		cocaine	3139.24	1554.21	440.62	2333.80	4062.49			
		alcohol	2762.84	604.03	176.52	2421.47	3115.40			
	happiness	control	1638.14	693.46	118.67	1419.30	1887.99	1.880	0.162	0.064
		cocaine	2002.53	759.48	212.33	1609.03	2429.88			
		alcohol	1968.14	520.40	148.46	1668.71	2267.35			
***BEAST***	anger	control	2002.88	1120.18	198.51	1649.21	2403.37	3.878	.027	.126
		cocaine	2503.75	1270.35	346.98	1842.25	3224.00			
		alcohol	3072.46	1140.17	324.36	2458.21	3718.09			
	fear	control	2334.25	1192.79	203.48	1960.63	2754.41	0.608	0.548	0.022
		cocaine	2472.77	618.26	168.94	2139.94	2779.54			
		alcohol	2739.86	1036.42	293.61	2176.09	3352.66			
	sadness	control	1906.22	810.26	145.01	1638.27	2221.24	3.974	0.025	0.128
		cocaine	2292.30	835.36	246.79	1834.02	2799.05			
		alcohol	2757.74	1217.09	353.51	2082.98	3522.77			
	happiness	control	2351.63	1695.36	310.28	1830.18	3052.76	2.016	0.143	0.072
		cocaine	3436.15	1838.36	505.16	2485.67	4458.77			
		alcohol	2719.00	1112.54	337.21	2077.23	3396.27			

*Note*: bootstrap estimation was used to derive 95% confidence intervals. Facial Emotion Recognition (FER); Body Emotion Recognition (BEAST); Standard Deviation (SD); Standard Error (SE); Confidence Interval (CI).

**Table 5:** Statistics, mean and standard deviation of accuracy for the disgust rating task.

		*MEAN*	*SD*	*Bootstrapped SE*	*Bootstrapped CI*		*F*	*SIG.*	*η* ^2^ *_P_*
***AMP***	control	5.70	1.58	0.27	5.09	6.19	0.081	0.923	0.003
	cocaine	5.48	2.16	0.60	4.22	6.58			
	alcohol	5.71	1.64	0.46	4.71	6.48			
***ANIM***	control	4.37	1.29	0.22	3.94	4.81	0.954	0.392	0.034
	cocaine	4.13	1.55	0.42	3.27	4.94			
	alcohol	4.86	1.32	0.38	4.12	5.60			
***PROD***	control	4.83	1.13	0.19	4.45	5.21	2.390	0.101	0.080
	cocaine	4.02	1.30	0.35	3.29	4.65			
	alcohol	4.83	1.18	0.35	4.19	5.53			
***CONT***	control	4.33	2.13	0.37	3.60	5.05	0.058	0.944	0.002
	cocaine	4.46	2.37	0.64	3.15	5.67			
	alcohol	4.58	2.45	0.70	3.15	5.94			
***DEATH***	control	5.88	1.41	0.24	5.38	6.33	0.039	0.962	0.001
	cocaine	5.98	0.98	0.28	5.39	6.50			
	alcohol	5.83	1.46	0.43	4.89	6.59			
***FOOD***	control	4.55	1.46	0.26	4.07	5.06	0.879	0.421	0.031
	cocaine	3.88	1.73	0.48	2.90	4.77			
	alcohol	4.40	1.55	0.44	3.54	5.28			
***HYG***	control	4.59	1.20	0.20	0.36	0.32	1.745	0.184	0.060
	cocaine	3.85	1.31	4.17	3.10	3.79			
	alcohol	4.40	1.13	4.98	4.55	5.02			

*Note*: bootstrap estimation was used to derive 95% confidence intervals. Food images (food), body product images (prod), animal images (anim), contamination images (cont), death images (death), envelope violation images (amp), hygiene images (hyg); Standard Deviation (SD); Standard Error (SE); Confidence Interval (CI).

**Table 6:** Statistics, mean and standard deviation of reaction times for disgust rating task.

		*Mean*	*SD*	*Bootstrapped SE*	*Bootstrapped CI*		*F*	*SIG.*	*η* ^2^ *_P_*
***AMP***	control	2382.70	1165.61	213.66	1982.44	2801.31	2.878	0.065	0.098
	cocaine	3615.45	2632.69	688.50	2379.98	5099.61			
	alcohol	3129.99	1242.15	352.91	2477.72	3867.78			
***ANIM***	control	3279.42	1873.84	326.04	2674.10	3954.13	0.521	0.597	0.019
	cocaine	3187.97	1089.49	311.25	2623.12	3838.37			
	alcohol	3762.13	657.00	190.06	3393.90	4138.15			
***PROD***	control	2844.33	1383.61	234.75	2417.96	3339.93	2.216	0.119	0.075
	cocaine	3637.15	1637.30	457.65	2758.13	4587.36			
	alcohol	3563.30	865.70	252.08	3070.80	4075.35			
***CONT***	control	3525.03	2593.14	452.04	2782.34	4540.96	0.098	0.906	0.004
	cocaine	3202.17	1271.96	369.09	2475.44	3904.07			
	alcohol	3424.83	1314.16	376.75	2719.68	4186.29			
***DEATH***	control	2768.74	1794.37	314.75	2178.50	3418.10	0.494	0.613	0.018
	cocaine	3353.15	2142.67	587.66	2258.69	4535.38			
	alcohol	2888.85	1313.09	380.32	2251.34	3750.64			
***FOOD***	control	3128.48	1467.45	256.01	2645.58	3685.81	3.807	0.028	0.124
	cocaine	3907.05	1511.11	430.97	3103.54	4777.37			
	alcohol	4433.06	1497.46	432.46	3635.11	5294.57			
***HYG***	control	2937.88	1113.78	189.88	2588.20	3319.74	1.277	0.287	0.045
	cocaine	3420.81	1348.07	394.82	2706.23	4267.60			
	alcohol	3401.43	747.77	220.36	2976.56	3834.86			

*Note*: bootstrap estimation was used to derive 95% confidence intervals. Food images (food), body product images (prod), animal images (anim), contamination images (cont), death images (death), envelope violation images (amp), hygiene images (hyg); Standard Deviation (SD); Standard Error (SE); Confidence Interval (CI).

**Table 7:** Statistics, mean and standard deviation of reaction times for inverse efficiency score (IES).

			*Mean*	*SD*	*Bootstrapped SE*	*Bootstrapped CI*		*F.*	*SIG.*	*η* ^2^ *_P_*
**FER**	anger	control	38.90	40.63	6.91	26.66	53.54	0.01	0.99	0.00
		cocaine	37.20	22.39	5.98	26.47	49.69			
		alcohol	38.21	13.96	3.94	30.63	46.50			
	fear	control	36.65	25.86	4.40	29.08	46.48	1.55	0.22	0.05
		cocaine	41.32	20.32	5.59	31.11	53.12			
		alcohol	51.51	27.24	7.53	37.42	67.05			
	sadness	control	47.94	84.93	14.67	24.59	81.53	0.24	0.78	0.01
		cocaine	43.47	32.91	9.05	28.04	63.34			
		alcohol	32.24	8.32	2.43	27.42	37.02			
	happiness	control	20.23	19.57	3.41	14.71	28.18	0.01	0.99	0.00
		cocaine	21.09	8.30	2.32	16.69	26.02			
		alcohol	20.33	6.56	1.91	16.82	24.05			
**BEAST**	anger	control	23.99	17.5	6.92	26.84	53.58	2.123	0.130	0.075
		cocaine	32.83	25.66	6.11	26.38	50.76			
		alcohol	37.42	20.61	3.97	30.80	46.24			
	fear	control	27.57	16.74	4.45	29.22	46.76	0.874	0.423	0.033
		cocaine	27.99	8.08	5.77	29.99	52.87			
		alcohol	34.60	18.30	7.92	36.85	67.91			
	sadness	control	21.49	8.94	15.07	25.05	83.81	4.591	0.014	0.148
		cocaine	27.55	10.50	9.04	27.66	63.77			
		alcohol	31.97	14.77	2.41	27.58	37.11			
	happiness	control	28.64	20.37	3.27	15.04	27.64	3.571	0.035	0.123
		cocaine	48.57	29.94	2.30	16.71	25.82			
		alcohol	33.57	17.31	1.92	16.64	24.18			

*Note*: bootstrap estimation was used to derive 95% confidence intervals. Standard Deviation (SD); Standard Error (SE); Confidence Interval (CI).

## Data Availability

The data are not publicly available due to ethical restrictions.
